# Practical Points in Nursing, for Nurses in Private Practice

**Published:** 1897-09

**Authors:** 


**Practical Points in Nursing, for Nurses in Private Practice.** By EMILY A. M. STONEY. Pp. 456. London: The Scientific Press, Limited. [N.D.]

Although this book bears an English imprint only, its authorship and publication are Transatlantic. As it is somewhat specialised in its aim, methods of improvising the usual nursing appliances are described and instructions given for the treatment of emergencies, and for the management of ordinary cases until the physician or surgeon arrives. But almost the whole of the book will prove equally useful to hospital and private nurses, and we can confidently recommend it to them as a good text-book on the theory and practice of nursing, and also as a handy book of reference. In addition to general and special nursing instructions, it contains a copious glossary, dose-list, various useful tables, and a section on elementary anatomy and physiology. This variety of contents, and the existence with it of a good index, are the features which lead us to pronounce the book handy for reference. The portions which deal more strictly with the art of nursing contain much sound advice.

